# Development of Chitosan-Carbon Dot Hybrid Nanoemulsomes for MEIS2 Inhibitor Delivery and Bioimaging in Colorectal Cancer

**DOI:** 10.3390/life16040591

**Published:** 2026-04-01

**Authors:** Buğra Onat, Deniz Özol, Seda Karakaş, Fatih Kocabaş

**Affiliations:** 1 Department of Genetics and Bioengineering, Faculty of Engineering, Yeditepe University, 34775 Istanbul, Türkiye; deniz.ozol@std.yeditepe.edu.tr (D.Ö.); seda.karakas@yeditepe.edu.tr (S.K.); 2 Department of Molecular Biology and Genetics, Faculty of Engineering and Natural Sciences, Istanbul Atlas University, 34408 Istanbul, Türkiye

**Keywords:** MEIS inhibitor delivery, hybrid nanoemulsome, colorectal cancer therapy

## Abstract

Homeobox protein MEIS2 has been strongly implicated in colorectal cancer (CRC) progression and metastatic potential, making its targeted inhibition a promising therapeutic strategy. However, recently developed MEIS inhibitors are limited by poor aqueous solubility, instability under physiological conditions, and insufficient intracellular accumulation, which restrict their clinical applicability. To overcome these challenges, a multifunctional hybrid nanoemulsome system was developed by integrating boron–silane-doped carbon dots (CDs) with chitosan via glutaraldehyde crosslinking, followed by emulsification with oleic acid and non-ionic surfactants (Span 80 and Tween 20/80) in the presence of a MEIS inhibitor (MEISi-2). The resulting composite exhibited high structural stability, excellent biocompatibility, and a drug encapsulation efficiency of 96.2%. Fourier-transform infrared spectroscopy (FTIR) and dynamic light scattering (DLS) analyses confirmed successful hybridization and the formation of nanoemulsions with an average particle size of approximately 320 nm following drug loading. The system demonstrated controlled drug release under physiological conditions. In vitro studies using HCT116 CRC and HaCaT healthy keratinocytes revealed effective cellular uptake and selective cytotoxicity. The intrinsic fluorescence properties of CDs enabled real-time monitoring of intracellular drug delivery via DAPI-channel imaging. Overall, this hybrid nanoemulsome platform provides a stable and efficient delivery system for MEIS inhibitors and represents a promising strategy for the treatment of CRC. Furthermore, this approach may be extended to other poorly soluble amphiphilic therapeutic agents.

## 1. Introduction

Colorectal cancer (CRC) is one of the most common malignancies worldwide [1]. It is characterized by a high metastatic potential, and MEIS2 plays a critical role in promoting cell migration and epithelial–mesenchymal transition (EMT). In CRC, MEIS2 has been identified as a pro-metastatic factor, where its overexpression is associated with decreased patient survival [2]. Proteomic analyses have further identified MEIS2 as a key regulator of aggressive proliferation, invasion, and poor prognosis in CRC [3]. Functional studies have demonstrated that MEIS2 knockdown suppresses migration, invasion, and EMT by modulating HOX–PBX signaling networks and downstream MAPK/ERK pathways [4,5]. Conversely, epigenetic repression of MEIS2 has also been linked to enhanced cancer stemness and tumor progression in CRC [6]. Collectively, these findings highlight MEIS2 as a clinically relevant therapeutic target in CRC.

Our recently developed MEIS inhibitor represents a promising small molecule that directly targets MEIS2 in vitro. However, due to its amphiphilic nature and limited aqueous solubility, it exhibits reduced bioavailability and limited translational potential [7]. These physicochemical limitations necessitate the development of advanced drug delivery systems to improve its stability and therapeutic efficacy.

Carbon dots (CDs) are versatile nanomaterials widely explored for drug delivery applications due to their excellent biocompatibility, tunable surface chemistry, and intrinsic photoluminescence properties, making them suitable theragnostic platforms [8,9]. Through surface functionalization, CDs can interact covalently or non-covalently with polymeric matrices, thereby enhancing structural stability and enabling controlled drug release and real-time tracking [10]. Among various biopolymers, chitosan is preferred for constructing hybrid nanocomposites due to its cationic nature and its ability to form cross-linked networks with improved solubility and stability [11].

In conventional CD synthesis approaches, amine groups are often introduced using ethylenediamine. In contrast, the present strategy deliberately limits surface amine density to minimize undesired crosslinking [12]. In this context, (3-aminopropyl) triethoxysilane (APTES) incorporation during CD synthesis enables the formation of Si–O–C and Si–O–Si linkages, reinforcing the structural framework of the CDs [13,14]. Additionally, boron doping modulates surface charge and electronic interactions, improving colloidal dispersibility. PEG4000 acts as a stabilizing and passivating agent during nucleation and growth, increasing surface C–O–C functionalities and enhancing aqueous solubility and colloidal stability [15,16]. This dual modification strategy preserves sufficient reactive groups on the CD surface, enabling efficient covalent conjugation with chitosan via glutaraldehyde crosslinking while preventing aggregation [17,18]. Consequently, this surface engineering approach preserves the photoluminescence properties of CDs while providing a stable platform for drug delivery applications [19].

Due to its amphiphilic structure, preliminary solubility studies revealed that MEISi-2 does not readily dissolve in commonly used organic solvents for polymeric (e.g., PLA/PLGA) or lipid-based nanoparticle systems, such as dichloromethane (DCM) and chloroform. This observation supports the rational design of a hybrid nanoemulsion system that accommodates the amphiphilic nature of MEISi-2 without relying on conventional solvent evaporation methods or halogenated solvents [20,21,22].

To address the poor aqueous solubility and salt-induced precipitation of MEISi-2 under physiological conditions, CD–chitosan hybrids were further emulsified with MEISi-2 dissolved in oleic acid using non-ionic surfactants, including Tween 20/80 and Span 80. In this configuration, the hydrophobic tails of oleic acid and Span 80 form a lipophilic core that encapsulates MEISi-2, facilitating its dispersion in the aqueous phase. The resulting nanoemulsion exhibits an oil-in-water (O/W) structure, where the oleic acid/Span 80 phase constitutes the internal oil phase containing the drug [23,24,25]. Tween 20 stabilizes the interfacial layer, enhances the solubility of CD–chitosan hybrids, and prevents droplet coalescence. Within this system, MEISi-2 is molecularly dispersed in the oil phase and stabilized through hydrophobic interactions and hydrogen bonding within the chitosan-CD network, contributing to overall colloidal stability [26,27].

This integrated structural design enables high encapsulation efficiency while preserving both fluorescence properties and colloidal integrity. Therefore, this study aims to develop a multifunctional hybrid nanoemulsion platform that combines organic–inorganic hybridization, surface silanization, and surfactant-assisted emulsification for the delivery and optical tracking of poorly soluble amphiphilic drugs, with a specific focus on MEIS inhibitors for CRC therapy [28].

## 2. Materials and Methods

### 2.1. Chemicals

Oleic acid (Thermo Scientific, Waltham, MA, USA), citric acid (Thermo Scientific, Waltham, MA, USA), Tween 20 (Thermo Scientific, Waltham, MA, USA), Span 80 (Thermo Scientific, Waltham, MA, USA), Tween 80 (Merck, Darmstadt, Germany), acetone (Isolab, Eschau, Germany), absolute ethanol (Isolab, Eschau, Germany), (3-aminopropyl)triethoxysilane (APTES) (Sigma-Aldrich, St. Louis, MO, USA), boric acid (Thermo Scientific, Waltham, MA, USA), glycerol (Isolab, Eschau, Germany), chitosan, EDTA, and acetic acid (Thermo Scientific, Waltham, MA, USA), propidium iodide (Invitrogen, Thermo Fisher Scientific, Waltham, MA, USA), and polyethylene glycol 4000 (PEG4000), (Merck, Darmstadt, Germany).

### 2.2. Synthesis of CD

Citric acid (1.25 g) (Thermo Scientific, Waltham, MA, USA), boric acid (0.01 g) Thermo Scientific, Waltham, MA, USA), glycerol (2.5 mL) (Isolab, Eschau, Germany), PEG4000 (0.125 g) (Merck, Darmstadt, Germany)., and APTES (10 μL) (Sigma-Aldrich, St. Louis, MO, USA) were dissolved in 10 mL of Milli-Q water (Milli-Q water purification system Merck Millipore, Darmstadt, Germany) and mixed until fully dissolved. The solvent was evaporated under reduced pressure at 70 °C. The resulting solidified samples were further heated at 200 °C for 6 h and subsequently redissolved in 10 mL of Milli-Q water (Milli-Q water purification system Merck Millipore, Darmstadt, Germany). Aggregates were removed by centrifugation at 10,000× *g*, followed by filtration through a 0.45 µm polyethersulfone (PES) filter (Sartorius, Göttingen, Germany) [29,30,31].

### 2.3. Synthesis of CDChitosan

Chitosan (2.5% *w*/*w*) (Thermo Scientific Waltham, MA, USA), and EDTA (0.25% *w*/*w*) (Thermo Scientific Waltham, MA, USA), were dissolved in 60 mL of 1% (*v*/*v*) acetic acid (Isolab, Eschau, Germany) solution under continuous stirring until fully solubilized. A 2.5% (*w*/*w*) CD solution was then added, and the mixture was stirred overnight. Aggregates were removed by filtration using a 0.45 µm polyethersulfone (PES) filter (Sartorius, Göttingen, Germany) filter. Subsequently, 60 μL of glutaraldehyde (25% aqueous solution) (Thermo Scientific, Waltham, MA, USA) solution was added, and the mixture was incubated under gentle stirring at 4 °C overnight. Following crosslinking, 30 mL of absolute ethanol (non-solvent) was added, and the mixture was stirred for 1 h at 4 °C. The resulting CD–chitosan samples were centrifuged at 14,000× *g* for 20 min and washed 3–4 times with Milli-Q (Milli-Q water purification system Merck Millipore, Darmstadt, Germany) water. The final product was resuspended in Milli-Q water (Milli-Q water purification system Merck Millipore, Darmstadt, Germany) containing 0.067% Tween 20 [32,33].

### 2.4. Emulsification of CDChitosanMEISi

MEISi-2 (4 mg), oleic acid (100 μL), Span 80 (200 μL), and Tween 80 (200 μL) were dissolved in a 10 mL ethanol/acetone mixture (1:1, *v*/*v*). The solvent was evaporated under reduced pressure with continuous stirring overnight to obtain the oil phase. The CD–chitosan dispersion was gradually added to the oil phase under high-speed stirring, and the system was mixed for 4 h at room temperature [34,35]. A control formulation was prepared using the same composition without MEISi-2. The resulting nanoemulsions were centrifuged at 14,000× *g* for 20 min and washed 3–4 times with Milli-Q (Milli-Q water purification system Merck Millipore, Darmstadt, Germany) water containing 0.005% Tween 20 (Thermo Scientific, Waltham, MA, USA). The final products were resuspended in 0.005% Tween 20 (Thermo Scientific, Waltham, MA, USA) and stored at 4 °C until further use. Prior to characterization and in vitro experiments, samples were diluted to the desired concentration and filtered through a 0.45 µm polyethersulfone (PES) filter (Sartorius, Göttingen, Germany) [36,37].

### 2.5. Characterization of CDChitotsanMEISi

CD surface chemistry was analyzed using FTIR spectroscopy (Nicolet iS50 FTIR spectrometer Thermo Fisher Scientific, Waltham, MA, USA), fluorescence spectroscopy, and UV–Vis absorbance measurements. Drug encapsulation efficiency was determined using a Varioskan LUX multimode microplate reader (Thermo Fisher Scientific, Waltham, MA, USA) at a dilution of 1:30. Particle size distribution and zeta potential measurements were performed using dynamic light scattering (DLS), (Zetasizer Nano ZS Malvern Instruments, Malvern, UK) at a dilution of 1:1000. Surface morphology was analyzed using atomic force microscopy (XE-100 Park Systems, Suwon, Republic of Korea). Drug release profiles were evaluated using a Varioskan LUX microplate reader in kinetic mode under continuous shaking at 300 rpm. Samples were diluted (1:30) in 1× PBS and complete DMEM (Gibco, Thermo Fisher Scientific, Waltham, MA, USA) and incubated for 48 h at room temperature.

### 2.6. Cell Culture Conditions

HaCaT (Cytion 300493 DSMZ, Braunschweig, Germany) and HCT116 (ATCC CCL-247 Manassas, VA, USA) cells were cultured in DMEM High Glucose (Gibco, Thermo Fisher Scientific, Waltham, MA, USA) supplemented with 10% heat-inactivated FBS (Gibco, Thermo Fisher Scientific, Waltham, MA, USA) and 1% penicillin–streptomycin (PAN-Biotech, Aidenbach, Germany) at 37 °C in a humidified atmosphere containing 5% CO_2_. Cells were passaged at ~80% confluency using 0.25% trypsin–EDTA (Multicell Technologies, Woerden, The Netherlands). For all experiments, CD–chitosan formulations were adjusted to provide equivalent MEISi-2 concentrations across treatment groups.

### 2.7. Drug Uptake Assay

Cells were seeded in 6-well plates at a density of 250,000 cells per well and incubated overnight. Cells were treated with 5 μM MEISi-2, CD–chitosan, or CD–chitosan–MEISi for 12 h. After treatment, cells were trypsinized and washed multiple times with 1× PBS. Cellular uptake was analyzed by using CytoFLEX flow cytometry (Beckman Coulter, Brea, CA, USA) using the DAPI channel, and at least 10,000 events were recorded per sample.

### 2.8. Cell Viability/Metabolic Activity Assay (MTS)

Cells were seeded in 96-well plates at a density of 7500 cells per well and incubated for 24 h. Cells were treated with various concentrations of MEISi-2, CD–chitosan, and CD–chitosan–MEISi for 24 and 48 h. Following treatment, cells were washed with 1× PBS. A 10% MTS (CellTiter 96^®^ AQueous One Solution Cell Proliferation Assay Promega, Madison, WI, USA) solution prepared in 1× PBS (Gibco, Thermo Fisher Scientific, Waltham, MA, USA) containing glucose (4.5 g/L) was added, and the plates were incubated for 2 h. Cell viability was assessed by measuring absorbance at 490 nm using a Varioskan LUX (Thermo Fisher Scientific, Waltham, MA, USA) microplate reader. The MTS assay reflects metabolic activity rather than direct clonogenic survival [38].

### 2.9. Cell Cycle Assay

Cells were seeded in 6-well plates at 250,000 cells per well and incubated for 24 h. Cells were treated with 5 μM MEISi-2, CD–chitosan, or CD–chitosan–MEISi for 24 h. Following treatment, cells were trypsinized and fixed with 70% ice-cold ethanol for 4 h. Cells were incubated with RNase A (Sigma-Aldrich, Merck, Darmstadt, Germany) (100 μg/mL) at 37 °C for 45 min, followed by staining with propidium iodide (Invitrogen, Thermo Fisher Scientific, Waltham, MA, USA) prior to analysis. Cell cycle distribution was analyzed using flow cytometry (CytoFLEX Beckman Coulter, Brea, CA, USA) with the PE channel, and at least 10,000 events were recorded per sample [39].

### 2.10. Colony Formation Assay

HCT116 cells were seeded in 6-well plates at a density of 5000 cells per well and allowed to attach for 24 h. Cells were treated with 5 μM MEISi-2, CD–chitosan, or CD–chitosan–MEISi for 24 h. Following treatment, fresh DMEM was added, and cells were incubated for 8 days. Colonies were fixed with paraformaldehyde (PFA, 4% aqueous solution) (ProSciTech, Thuringowa Central, QLD, Australia) for 15 min at 4 °C and stained with crystal violet (Sigma-Aldrich, Merck, Darmstadt, Germany) for 20 min at room temperature. Wells were washed with PBS, and colony formation was analyzed.

### 2.11. Data Analysis/Statistical Analysis

Data are presented as mean ± standard deviation (SD). Graphical representations were generated using Python 3 (Anaconda distribution, Python Software Foundation, USA) Statistical analyses were performed using GraphPad Prism (version 10.4.1, GraphPad Software, San Diego, CA, USA) Group comparisons were conducted using one-way analysis of variance (ANOVA) followed by Dunnett’s multiple comparisons test to compare each treatment group with the control. A *p* value < 0.05 was considered statistically significant.

## 3. Results

### 3.1. Characterization of CDBA and CDBAAPT

**Figure 1:**
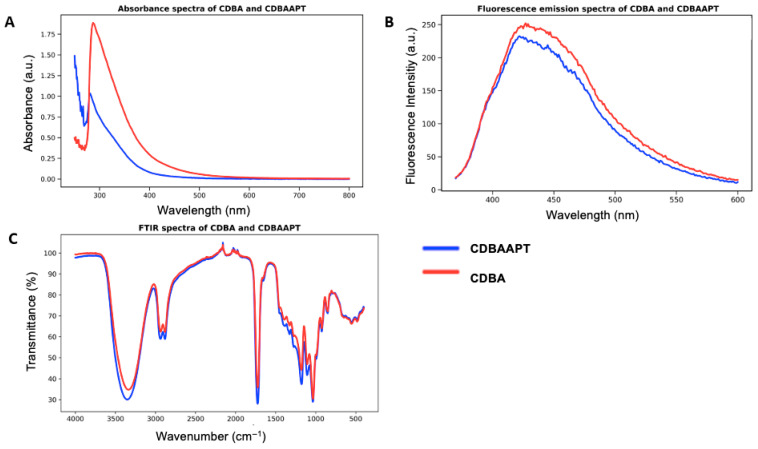
(**A**) Absorbance spectrum of CDBA and CDBAAPTES systems. (**B**) Fluorescence emission spectra of CDBoricAcid (CDBA) and CDBoricAcidAPTES (CDBAAPT) system. (**C**) FTIR spectrum comparison of CDBA and CDBAAPT systems.

The CD synthesis was optimized to achieve strong blue photoluminescence emission, as the excitation/emission window is required for DAPI-channel visualization [40]. Pre-synthesized CDs exhibited emission within the DAPI region, as shown in Figure 1A,B.

APTES and boric acid (BA) were introduced to promote surface functionalization through the formation of stable silane-containing structures. In Figure 1C, broad O–H stretching vibrations at 3200–3500 cm^−1^ and a sharp peak at 1730 cm^−1^, corresponding to C=O stretching, indicate a COOH-rich surface. The presence of Si–O–Si stretching vibrations at 1000–1100 cm^−1^ and Si–C stretching bands at 800–850 cm^−1^ confirms the successful incorporation of silane-based functionalization on the CD surface [41,42,43].

C–O vibrations observed in the 1050–1250 cm^−1^ region correspond to PEG and glycerol-based backbones. The stabilizing effect of PEG promotes uniform particle growth and reduces aggregation [16]. Furthermore, Figure 1C demonstrates that APTES introduction leads to the formation of an organized silane network. Surface modification was further enhanced by boron doping, which contributes to partial modulation of C–O and C–N groups through Si–C interactions [44]. The modified CD structures differ from pristine CDs due to the incorporation of boron and silane functionalities. These modifications play a critical role in inhibiting glutaraldehyde (GA)-induced aggregation, ensuring colloidal stability, and preserving functional surface integrity upon chitosan conjugation [45,46].

### 3.2. CDChitosanMEISi Characterization Results

**Figure 2:**
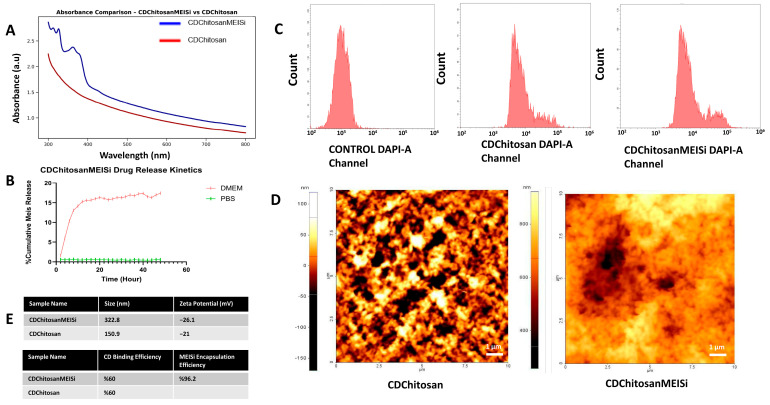
(**A**) Absorbance spectrum analysis CDChitosan and CDChitosanMEISi system. (**B**) CDChitosanMEISi drug release kinetics of 48 h treatment. (**C**) Drug uptake assay flow cytometry analysis at DAPI channel. The red histogram represents fluorescence intensity distribution of DAPI-positive cells. (**D**) AFM image of CDChitosan and CDChitosanMEISi system. (**E**) DLS and Zeta potential Results of CDChitosan and CDChitosanMEISi System.

Encapsulation efficiency of CDChitosan and CDChitosanMEISi systems was evaluated using UV–Vis and fluorescence spectroscopy. CDs were covalently incorporated into the chitosan matrix with an efficiency of approximately 60% (Figure S1) [47]. MEIS inhibitor (MEISi-2) exhibits characteristic absorption peaks at 350–410 nm, representing a distinct molecular signature within the composite system. Following emulsification, encapsulation efficiency reached 96.2%, indicating a strong affinity between MEISi-2 and the CDChitosan hybrid system (Figure 2A) [48].

The observed decrease in fluorescence intensity after emulsification may be attributed to high optical density-induced attenuation (inner filter effects) and increased light scattering within the nanoemulsion matrix (Figure S2) [49]. Nanoemulsified CDChitosan and CDChitosanMEISi systems form a highly viscous matrix, which prevents premature drug release. Intracellular fluorescence was detected in the DAPI channel via flow cytometry (Figure 2C), confirming effective cellular internalization [50]. While MEISi-2 incorporation does not significantly alter fluorescence emission, intracellular signal detection depends on cellular uptake and subsequent degradation of the chitosan matrix. UV–Vis and fluorescence analyses indicate that cellular uptake is largely completed within 24 h post-treatment (Figures S3 and S4).

Encapsulation efficiency is influenced by MEISi-2 loading due to its strong intramolecular interactions and hydrogen bonding capacity. The average particle size of CDChitosan nanoemulsions was 150.9 nm (Figure 2E), which increased to 322.8 nm upon MEISi-2 loading. This increase can be attributed to drug incorporation into the oleic acid core and slight swelling of the chitosan shell in aqueous media [51]. The amphiphilic nature of MEISi-2 promotes detergent-like molecular organization through hydrophobic and hydrogen-bonding interactions, contributing to enhanced formulation stability. Zeta potential decreased to approximately −5 mV upon MEISi-2 loading, suggesting the formation of a more compact and electrostatically balanced system. AFM analysis further confirmed morphological changes upon drug loading. CDChitosanMEISi films (Figure 2D) exhibited more heterogeneous and aggregated domains compared to CDChitosan, reflecting structural reorganization following MEISi-2 incorporation [33].

CDChitosanMEISi systems remained stable in PBS, showing negligible drug release (Figure 2B). In contrast, in complete DMEM containing serum, approximately 30% of MEISi-2 was released within 48 h. This indicates controlled diffusion under physiological conditions. The controlled release behavior is attributed to drug entrapment within the oleic acid core and diffusion-limited transport through the chitosan outer shell [34].

### 3.3. In Vitro Anti-Cancer Analysis of CDChitosanMEISi

**Figure 3:**
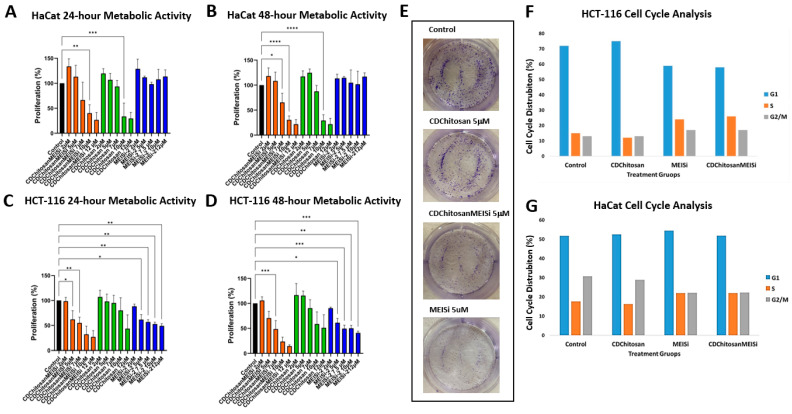
(**A**) HaCaT 24 h metabolic activity (MTS assay) results with Control, CDChitosanMEISi, CDChitosan, and MEIS inhibitor only. (**B**) HaCaT 48 h metabolic activity (MTS assay) results with Control, CDChitosanMEISi, CDChitosan, and MEIS inhibitor only. (**C**) HCT-116 24 h metabolic activity (MTS assay) results with Control, CDChitosanMEISi, CDChitosan, and MEISi-2 only. (**D**) HCT-116 48 h metabolic activity (MTS assay) results with Control, CDChitosanMEISi, CDChitosan, and MEISi-2 only. (**E**) HCT 116 Colony formation results with Control, CDChitosanMEISi, CDChitosan, and MEISi only. (**F**) HCT 116 cell cycle results with Control, CDChitosanMEISi, CDChitosan, MEIS inhibitor only. (**G**) HaCaT cell cycle results with Control, CDChitosanMEISi, CDChitosan, and MEISi-2 only. Statistical significance was determined using one-way ANOVA followed by Dunnett’s multiple comparisons test versus the control group (* *p* < 0.05, ** *p* < 0.01, *** *p* < 0.001, **** *p* < 0.0001).

Metabolic activity (MTS) assays were performed in HCT116 CRC cells and healthy HaCaT keratinocytes (Figure 3A,B). In HCT116 cells, a significant reduction in metabolic activity was observed at 5 μM for both free MEIS inhibitor and CDChitosanMEISi treatments. In contrast, the same concentration was well tolerated in HaCaT cells. However, at concentrations above 7 μM, increased cytotoxicity was observed in both MEISi-2-loaded and free drug groups [5,52]. This effect may be attributed to the amphiphilic nature of the chitosan-based nanoemulsion system, which can enhance membrane permeability at higher doses [53].

Cell cycle analysis revealed that MEISi-2 induces S/G2 phase arrest in HCT116 cells (Figure 3F,G). CDChitosanMEISi exhibited a similar cell cycle profile, confirming that the observed anti-proliferative effects are primarily driven by MEIS inhibition. Given the established role of MEIS2 in epithelial–mesenchymal transition (EMT) regulation in CRC, colony formation assays further demonstrated that MEIS inhibition significantly suppresses cancer stem cell-like properties in HCT116 cells. Importantly, the empty nanocarrier exhibited a profile like the untreated control group, confirming that the observed biological effects originate from MEISi-2 rather than carrier-related toxicity. Overall, both MEISi-2 and CDChitosanMEISi treatments significantly reduced long-term proliferative capacity and self-renewal potential in HCT116 cells [5].

## 4. Discussion and Conclusions

MEIS family proteins function as transcriptional regulators that control gene expression associated with cell cycle progression, epithelial–mesenchymal transition (EMT), and cancer stem cell-like properties. Depending on their expression level and cellular context, MEIS proteins may exert oncogenic effects in CRC [54]. Dysregulation of MEIS plays a critical role in CRC progression. In particular, MEIS2 expression has been strongly associated with stage II and III CRC, where it promotes metastasis and has been proposed as a potential biomarker. Moreover, MEIS2 has been shown to enhance proliferation and clonal expansion in CRC cell lines such as HCT116 and SW480. In the present study, similar biological outcomes were observed following treatment with both MEISi-2 and CDChitosanMEISi in HCT116 cells, providing functional validation of both the MEIS inhibitor and the designed delivery system [16]. Furthermore, previous studies have demonstrated that MEIS2 accelerates the G1/S phase transition in CRC cells, thereby promoting proliferation. Consistent with these findings, our results (Figure 3D) indicate that MEIS inhibition suppresses cell cycle progression, confirming effective disruption of MEIS-mediated signaling pathways [55].

MEISi-2 possesses a molecular structure comparable to hydrophobic drugs such as curcumin and paclitaxel; however, it undergoes salt-induced precipitation under aqueous ionic conditions, leading to the formation of poorly soluble aggregates. To overcome this limitation, a chitosan-decorated nanoemulsion system was employed. A similar emulsion-based strategy has been reported in literature where the R848 molecule—sharing comparable amphiphilic and low-solubility characteristics—was successfully stabilized within a nanoemulsion system. Both R848 and MEISi-2 exhibit amphiphilic behavior and limited aqueous solubility, making them susceptible to degradation in aqueous environments. In agreement with previous findings, our nanoemulsion design effectively protected MEISi-2 from degradation while enabling efficient cellular uptake [34].

In carbon dot synthesis, APTES-assisted silanization combined with boric acid-mediated boron doping results in a structurally reinforced surface architecture. The incorporation of Si–C and Si–O–Si bonds provides a physicochemically favorable interface for chitosan integration while enhancing electrostatic stabilization [56,57]. In addition, silane modification minimizes aggregation during glutaraldehyde (GA)-mediated crosslinking and enables the formation of a stable, water-soluble intermediate platform prior to emulsification [58]. Notably, silane modification did not significantly alter the fluorescence emission profile of CDs but primarily affected surface architecture and aqueous dispersibility. Similar findings have been reported for silane-functionalized CDs, where optical properties remain preserved despite significant changes in surface chemistry [58].

Chitosan-decorated nanoemulsion systems are known to form stable structures without phase separation during drug transport, as demonstrated by Khan et al. In our system, the observed zeta potential values (−21 mV for CDChitosan and −26.1 mV for CDChitosanMEISi) reflect the combined influence of non-ionic surfactants (Tween 20/80) and drug incorporation on surface charge [35]. Previous studies have shown that carbon dots can improve dispersion homogeneity and reduce aggregation in hybrid nanomaterial systems through enhanced surface functionalization and interfacial interactions [59]. The controlled release behavior observed in this study—approximately 30% drug release in serum-containing medium over 48 h—aligns with previously reported nanoemulsion-based sustained-release systems [60]. Upon MEISi-2 loading, an increase in hydrodynamic diameter was observed, which can be attributed to the amphiphilic nature of the drug and enhanced interfacial interactions within the hybrid nanoemulsion structure. Such size increases are commonly reported in polymeric and hybrid nanoemulsion systems and are typically associated with interfacial reorganization and hydrodynamic swelling [32]. Although increased particle size may moderately influence EPR-mediated tumor targeting efficiency, nanoparticles within the 200–400 nm range can still achieve effective tumor accumulation, particularly in highly permeable tumor microenvironments [61,62].

Fluorescence analysis confirmed that both the CDChitosan and CDChitosanMEISi systems exhibit detectable emission in the DAPI channel, indicating successful cellular association. Importantly, similar fluorescence intensity observed in CDChitosan without MEISi-2 supports the conclusion that the emission originates primarily from the carbon dot component [63].

Overall, this study demonstrates that the hybrid CDChitosanMEISi system provides an efficient and stable delivery platform for MEISi-2. The system maintains structural integrity, enables selective cytotoxicity, and supports controlled intracellular drug release. These findings highlight the potential of this multifunctional nanoemulsion-based platform as a therapeutic carrier for CRC treatment. A limitation of the present study is the lack of in vivo biodistribution analysis, which is essential to confirm tumor targeting and systemic stability. Future studies should focus on in vivo tumor accumulation, immune interactions, and real-time imaging of the system. Furthermore, this platform may be extended as a general delivery strategy for other poorly soluble amphiphilic therapeutic agents.

## Data Availability

The original contributions presented in this study are included in the article/Supplementary Materials. Further inquiries can be directed to the corresponding authors.

## Supplementary Materials

The following supporting information can be downloaded at: https://www.mdpi.com/article/10.3390/life16040591/s1. Figure S1. CD encapsulation efficiency analysis. Figure S2. CDChitosan CDChitosanMEISi fluorescence measurement. Figure S3. UV-Vis absorbance spectrum of MEISi-2 in DMSO. Figure S4. Fluorescence emission graph of the MEISi-2 inhibitor analyzed in aqueous medium.
