# Supplementary material for: Development of Chitosan-Carbon Dot Hybrid Nanoemulsomes for MEIS2 Inhibitor Delivery and Bioimaging in Colorectal Cancer
Source: Life (Basel). 2026 Apr 1;16(4):591. doi: 10.3390/life16040591 (PMC13117824; doi:10.3390/life16040591)
Supplement: Supplementary file 1 [file life-16-00591-s001.zip › life-4150399-supplementary.pdf]

Supplementary Figures and Figure Legends

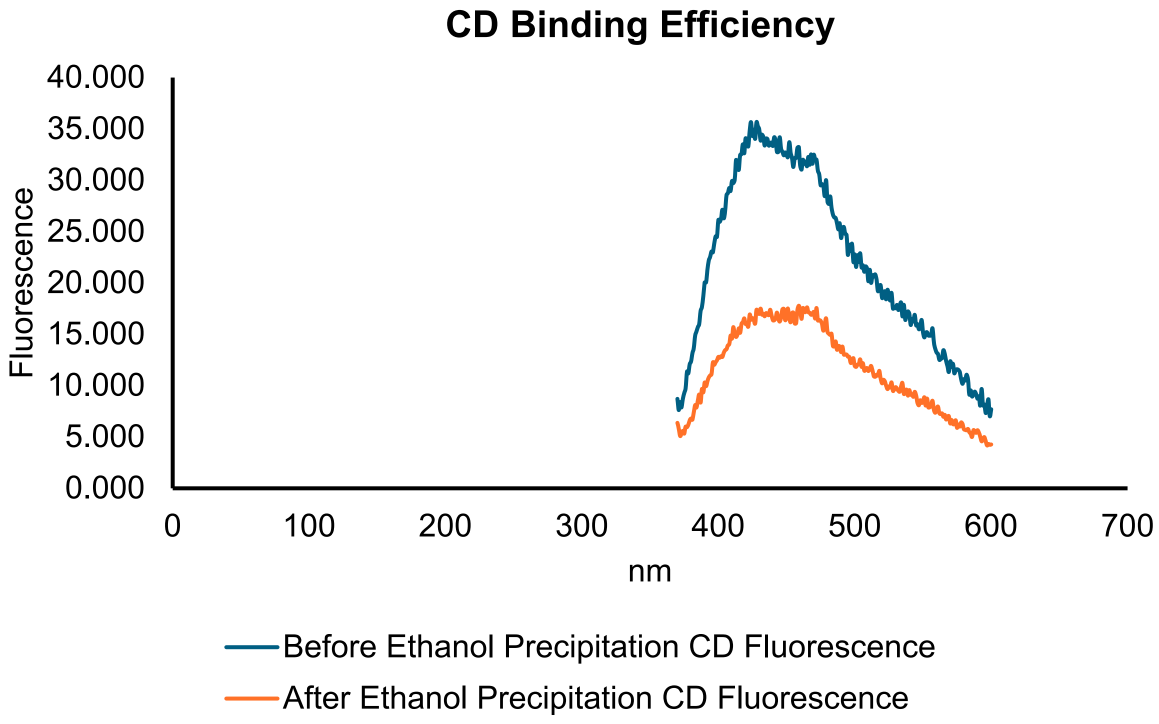

Figure S1. CD encapsulation efficiency analysis. Fluorescence spectra of carbon dots before and after ethanol precipitation were analyzed. Note that a decrease in fluorescence intensity after purification indicates removal of free carbon dots and precursors in the environment and confirms that successful attachment of CD in chitosan matrix.

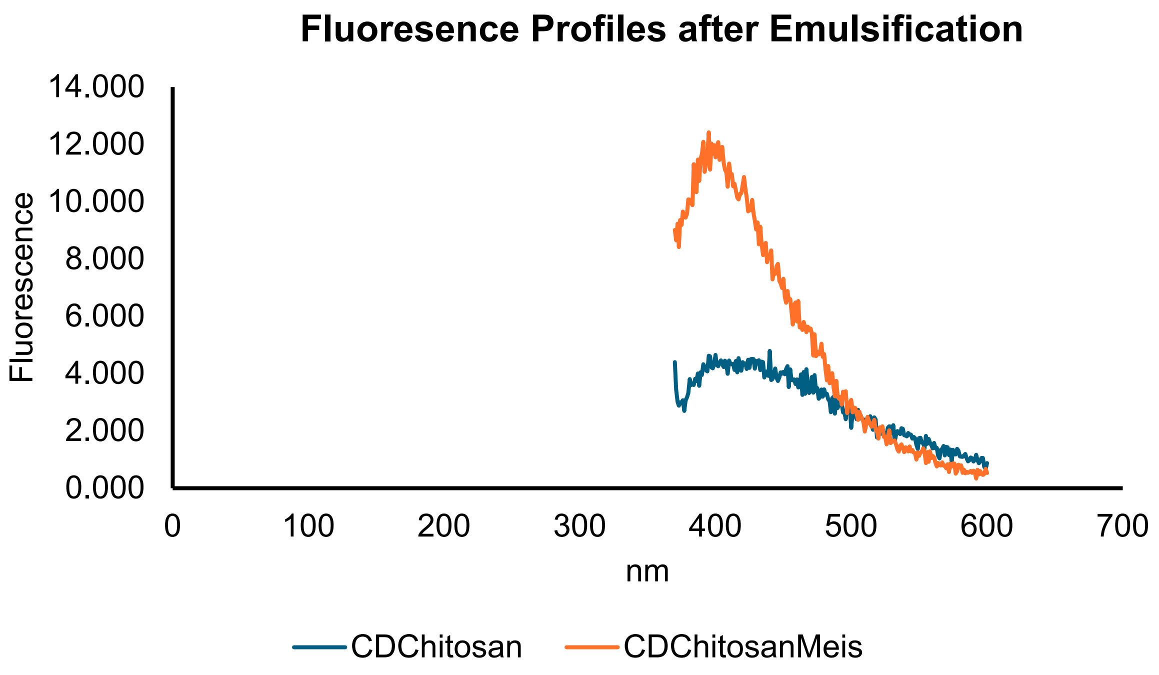

Figure S2. CDChitosan CDChitosanMEISi fluorescence measurement. Note that Fluorescence specta of CDChitosan and CDChitosanMEISi after emulsification MEISi-2 addition does not alter the characteristics of the CD in the enveloped system and there is no significant MEISi-2 characteristics fluorescence observed after emulsification.

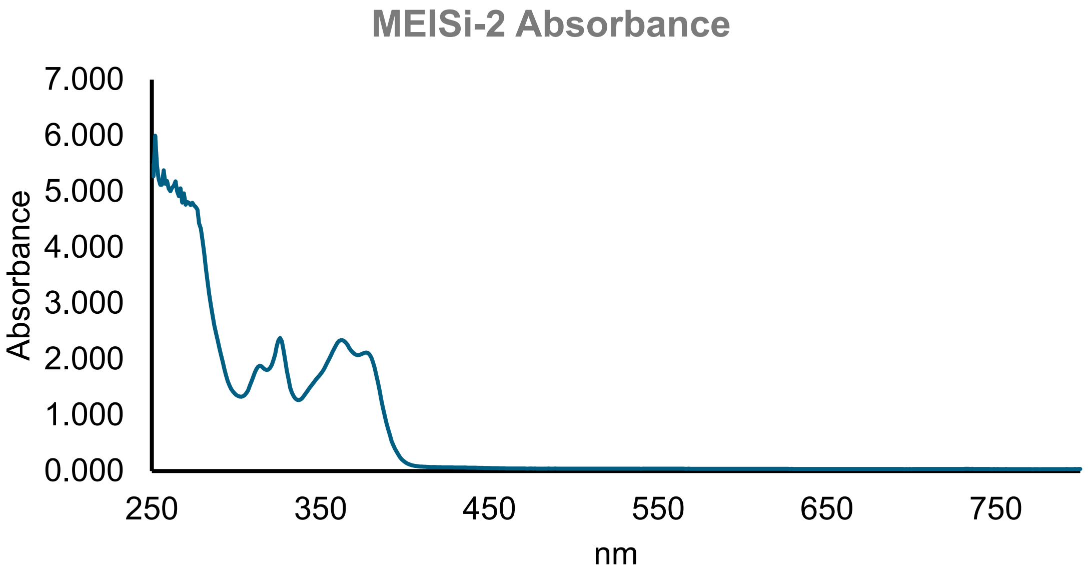

Figure S3. UV-Vis absorbance spectrum of MEISi-2 in DMSO. The absorbance profile of MEISi-2 shows the characteristic absorption bands of MEISi-2 at approximately 360 nm and 380 nm. This figure confirms that the inhibitor has optically distinctive properties.

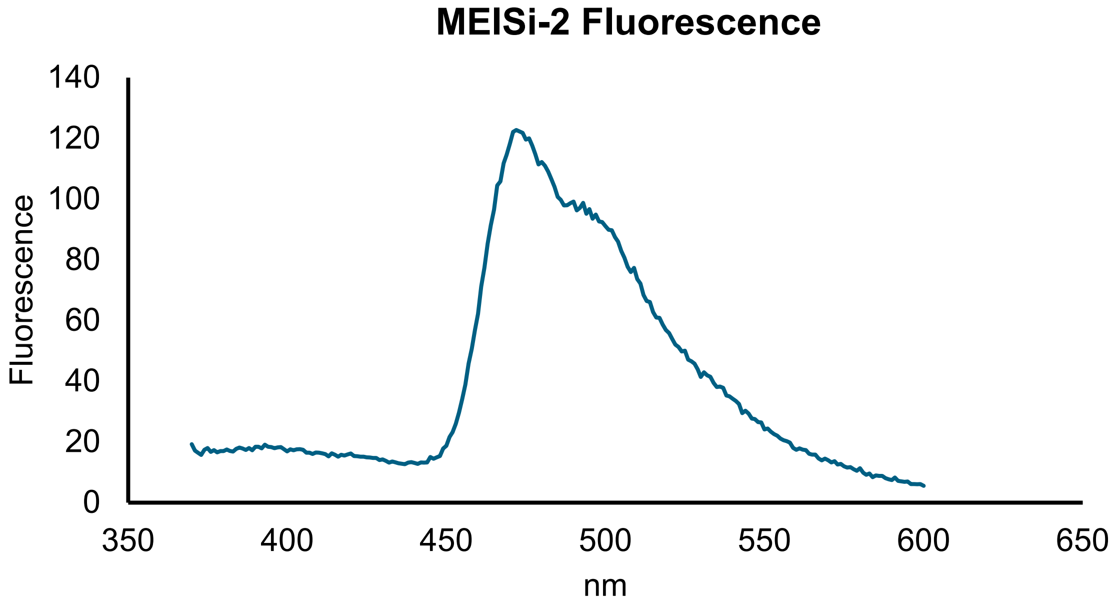

Figure S4. Fluorescence emission graph of the MEISi-2 inhibitor analyzed in aqueous medium. It was observed that MEISi-2(aq) excited at 370nm showed a strong fluorescence response with a peak at approximately 460nm. This feature is a defining optical property of MEISi-2.
